# Postreperfusion Blood Pressure Variability After Endovascular Thrombectomy Affects Outcomes in Acute Ischemic Stroke Patients With Poor Collateral Circulation

**DOI:** 10.3389/fneur.2019.00346

**Published:** 2019-04-12

**Authors:** Jun Young Chang, Sang-Beom Jeon, Cheolkyu Jung, Dong Seok Gwak, Moon-Ku Han

**Affiliations:** ^1^Department of Neurology, Asan Medical Center, Seoul, South Korea; ^2^Department of Radiology, Seoul National University Bundang Hospital, Seongnam, South Korea; ^3^Department of Neurology, Kyungpook National University Hospital, Daegu, South Korea; ^4^Department of Neurology, Seoul National University Bundang Hospital, Seongnam, South Korea; ^5^Department of Neurology, College of Medicine, Seoul National University, Seoul, South Korea

**Keywords:** computed tomography angiography, thrombectomy, stroke, blood pressure, collateral circulation

## Abstract

**Background and Purpose:** We evaluated the effect of 24 h blood pressure variability (BPV) on clinical outcomes in acute ischemic stroke patients with successful recanalization after endovascular recanalization therapy (ERT).

**Methods:** Patients with anterior circulation occlusion were evaluated if they underwent ERT based on multiphase computed tomography angiography and achieved successful recanalization (≥thrombolysis in cerebral ischemia 2b). Collateral degrees were dichotomized based on the pial arterial filling score, with a score of 0–3 defined as a poor collateral status. BPV parameters include mean, standard deviation, coefficient of variation, and variation independent of the mean (VIM) for systolic blood pressure (SBP), diastolic blood pressure (DBP), mean blood pressure, and pulse rate (PR). These parameters were measured for 24 h after ERT and were analyzed according to occlusion sites and stroke mechanisms. Associations of BPV parameters with clinical outcomes were investigated with stratification based on the baseline collateral status.

**Results:** BPV was significantly different according to the occlusion sites and stroke mechanisms, and higher BPV was observed in patients with internal carotid artery occlusion or cardioembolic occlusion. After adjustment for confounders, most BPV parameters remained significant to predict functional outcomes at 3 months in patients with poor collateral circulation. However, no significant association was found between BPV parameters and clinical outcomes in patients with good collateral circulation.

**Conclusion:** Postreperfusion BP management by decreasing BPV may have influence on improving clinical outcome in cases of poor collateral circulation among patients achieving successful recanalization after ERT.

## Introduction

Endovascular recanalization therapy (ERT) has been adopted as standard stroke care in patients with acute ischemic stroke (1–6). Time to recanalization and degree of recanalization are the most important predictors of clinical outcomes after ERT (7). Before recanalization, an effort to reduce the time from symptom onset to reperfusion is critical for penumbral salvage. After recanalization, an effort to control BP is important for penumbral salvage and inhibition of hemorrhagic transformation.

Adequate blood pressure management after recanalization is important for reducing reperfusion injury and improving outcomes in patients with acute ischemic stroke. Both higher and lower baseline systolic blood pressures (SBPs) are associated with poor outcomes in patients with acute ischemic stroke after ERT (8). Because of impaired cerebrovascular autoregulation after ischemic stroke, BP fluctuation directly affects the ischemic brain tissue, leading to the growth of the infarct core and poor functional outcomes (9).

Increased BPV is associated with sympathetic overactivity, hyperglycemia, immunosuppression, and coagulopathy (10). Prior studies have indicated that high BPV is associated with poor neurological outcomes in acute ischemic stroke patients not indicated for ERT, non-recanalized patients after intravenous thrombolysis (IVT) for ICA occlusion, or patients with incomplete recanalization after ERT (9, 11–13). The effect of BPV on the clinical outcomes in recanalized patients after ERT had not been evaluated before.

A paucity of studies has investigated postreperfusion BPV and clinical outcomes after ERT. In addition, data are lacking whether the effects of BPV on clinical outcomes are different in patients with different baseline collateral statuses. In this study, we evaluated the effect of 24 h BPV on clinical outcomes in the acute ischemic stroke patients with successful recanalization after ERT.

## Methods

We retrospectively selected patients from the database at a single hospital-based stroke registry. The hospital institutional review board approved this retrospective study (B-1705-399-103). This study included patients with anterior circulation occlusion who underwent ERT based on multiphase CT angiography (CTA) and achieved successful recanalization (thrombolysis in cerebral ischemia [TICI] ≥ 2b) at the institute between April 2015 and February 2017. Baseline demographic and clinical information, including neurological outcomes and imaging data, were obtained from the registry. Patients who underwent ERT because of posterior circulation occlusion or bilateral occlusion were excluded from the analysis.

Multiphase CTA imaging was performed using a 256-slice multi-detector CT scanner (Brilliance iCT; Philips) equipped with a collimator of 128 × 0.625 mm, 120 kV, and 90 mAs. The first phase of multiphase CTA was acquired from the aortic arch to the vertex during the peak arterial phase. After a 4-s delay, patients were scanned from the skull base to the vertex for 3 s in the second phase (during the peak venous phase) and the third phase (during the late venous phase). A total of 90 mL of contrast agent was injected at a rate of 5 mL/s.

The collateral status was evaluated by pial arterial filling in the symptomatic hemisphere compared with the unaffected hemisphere. A 6-point scale was developed to assess collateral circulation in terms of the phase delay, vascular extent, and vascular prominence. The scoring system was validated with good reliability in a previous study by Menon et al. (14). The collateral score was established using a two-reader consensus. Collateral degrees were dichotomized based on the pial arterial filling score. A score of 0–3 indicates a poor collateral status, and a score of 4–5 indicates a good collateral status.

BP was measured every 15 min for 2 h; then, every 30 min for 6 h; and subsequently, every hour for 24 h after IVT. In the absence of any clinical event, BP was evaluated at least once every hour in the non-paretic arm of the patient in the stroke and intensive care units. A non-invasive electronic monitoring device that automatically recorded the measurements in the electronic medical record was used. Acute BP measurement and management followed the current guidelines proposed by the American Stroke Association and the Korean Stroke Society (15). Before recanalization, blood pressure was not lowered unless exceeds 220 mmHg for SBP and 120 mmHg for diastolic blood pressure (DBP). If indicated for IVT, blood pressure was controlled under 185 mmHg for SBP and 110 mmHg for DBP. Once recanalized successfully after ERT, SBP was controlled over 100 mmHg and below 160 mmHg. A method and a level of BP control was left to an attending clinician's decision.

We calculated the following parameters of SBP, DBP, mean BP (MBP), and pulse rate (PR) 24 h after admission: mean, standard deviation (SD), coefficient of variation (CV, SD/mean), and variation independent of the mean (VIM). SD and CV were frequently used and validated in previous studies to measure BPV (11, 16, 17). Since SD and CV are positively correlated with mean BP, it is necessary to derive a supplemental variable uncorrelated with mean levels. VIM is a transformation of SD and is devised to be independent of mean. VIM is calculated as *k* × SD/(mean), where ß is derived from the regression coefficient of the plot of the natural SD logarithm (y-axis) on the natural mean logarithm (x-axis) (18). We corrected VIM using the following equation:

Corrected VIM=(VIM× mean of CV)/meanof VIM

Early neurological improvement (ENI) is defined as ≥50% or as an 8-point reduction from the baseline in National Institutes of Health Stroke Score (NIHSS). A certified neurologist determined the neurological status of patients on days 1 and 7 using NIHSS. For patients discharged prior to 7 days post-injury, the neurological assessment was performed at the day of discharge using NIHSS. Functional outcomes were assessed using modified Rankin scale (mRS) by a neurologist at the outpatient clinic or by a trained nurse if a follow-up visit was not available after 3 months. A favorable outcome was defined as modified Rankin scale score (mRS) ≤ 2.

### Statistical Analyses

Baseline characteristics (age, sex, initial stroke severity, stroke risk factors, stroke mechanism, occlusion site, onset to recanalization time, and clinical outcome variables) were integratedly analyzed with the collateral circulation status and functional outcome. Median or mean values of BPV parameters were compared according to occlusion site, stroke mechanism, baseline collateral status, and functional outcome. Chi-squared or Fisher's exact test was used to analyze categorical variables; independent *t*-test, or Mann–Whitney *U*-test was used to analyze continuous variables. To estimate odds ratio (OR) and 95% confidence interval (CI) for the independent association of each BPV parameter with clinical outcomes, ordinal logistic regression analysis was performed. Because there was a significant interaction between BPV parameters and the collateral status, the analysis was performed with stratification based on the collateral status. Variables for adjustments were selected based on univariate analysis with a *p* ≤ 0.05. Among the selected variables (age, sex, initial NIHSS, DM, current smoking, and occlusion site), variables that did not contribute to the model were excluded using a likelihood ratio test. As a result, age, sex, initial NIHSS, and occlusion site were included in the final model. Goodness-of-fit was evaluated using the Hosmer–Lemeshow test. The predictive logistic model was internally validated by bootstrap simulations. Analyses were performed using Stata version 13.0 (StataCorp, Austin, TX, USA). All tests were 2-sided, and a *p* ≤ 0.05 was considered significant.

## Results

A total of 90 patients with acute ischemic stroke due to anterior circulation occlusion, who achieved successful recanalization (≥TICI 2b), were included in the analysis. The median number of BP measurements was 31 (interquartile range, 26–37) and mean time from IAT to admission was 63.5 ± 46.6 min.

The mean age of the study subjects was 72.3 ± 11.8 years, and 60.0% of subjects were men. The initial median systolic blood pressure was 149.0 (interquartile range, 134.0–166.0) mmHg. The median baseline NIHSS score was 14.7 ± 5.5, and atrial fibrillation (51, 58.0%) was the most common risk factor in the patients. The occlusion sites were presented in order of frequency as follows: M1 segment of middle cerebral artery (MCA) (49, 54.4%); internal carotid artery (ICA) (26, 28.9%); and M2 segment of MCA (15, 16.7%). Anterior cerebral artery occlusion was noted in three patients, all of whom had tandem occlusion of the MCA. Tandem occlusion of ICA and MCA was found in four patients. The median time from symptom onset to recanalization was 216.0 min (interquartile range, 142.0–317.0). The mean SBP during 24 h after admission was 128.8 ± 11.9. Parenchymal hemorrhage (19) was observed in 16 patients (17.8%).

In addition, 40 patients (44.4%) had ENI on day 1, while 60 patients (66.7%) showed ENI on day 7 or discharge; 49 patients (54.4%) had poor collateral circulation. Patients with poor collateral circulation had a significantly higher baseline NIHSS, were more likely to have coronary artery disease, and were less likely to have a smoking habit (Table 1). Moreover, patients with poor collateral circulation were less likely to have ENI at days 1 and 7 and favorable outcomes at month 3 (Table 1).

**Table 1:** Baseline characteristics of the study subjects based on the collateral status and functional outcome.

	**Total**	**Baseline collateral status**			**Functional outcome at 3 month**		
		**Poor collateral (0–3), *n* = 49**	**Good collateral (4–5), *n* = 41**	***P*-value**	**Poor outcome (mRS >2), *n* = 41**	**Good outcome (mRS ≤ 2), *n* = 49**	***P*-value**
**DEMOGRAPHICS**							
Age, y, mean ± SD or median (IQR)	72.3 ± 11.8	74.8 ± 11.1	69.3 ± 12.1	0.03	78.0 (72.0–85.0)	68.0 (60.0–76.0)	<0.01
Male, *n*(%)	54 (60.0)	27 (55.1)	27 (65.9)	0.41	18 (43.9)	36 (73.5)	0.01
Initial systolic blood pressure at ER, median (IQR)	149.0 (134.0–166.0)	145.0 (133.0–164.0)	152.0 (137.0–168.0)	0.55	154.0 (138.0–178.0)	147.0 (132.0–164.0)	0.18
Initial NIHSS, mean ± SD	14.7 ± 5.5	17.1 ± 4.4	11.8 ± 5.3	<0.01	17.2 ± 4.7	12.6 ± 5.4	<0.01
NIHSS at 24 h after admission, median (IQR)	8.0 (4.0–15.0)	11.0 (6.5–20.0)	4.0 (1.5–9.5)	<0.01			
**RISK FACTORS**							
Previous stroke	23 (25.8)	15 (30.6)	8 (20.0)	0.37	9 (22.0)	14 (29.2)	0.6
DM, *n* (%)	25 (28.7)	17 (35.4)	8 (20.5)	0.2	17 (42.5)	8 (17.0)	0.02
HT, *n* (%)	43 (49.4)	27 (57.4)	16 (40.0)	0.16	24 (61.5)	19 (39.6)	0.07
Af, *n* (%)	51 (58.0)	32 (65.3)	19 (48.7)	0.18	25 (61.0)	26 (55.3)	0.75
Smoking, *n* (%)	21 (24.1)	6 (12.8)	15 (37.5)	0.02	4 (10.3)	17 (35.4)	0.01
Dyslipidemia, *n* (%)	17 (19.5)	8 (17.0)	9 (22.5)	0.71	7 (17.9)	10 (20.8)	0.95
CAD, n (%)	10 (11.6)	9 (19.1)	1 (2.6)	0.04	8 (20.5)	2 (4.3)	0.05
**TOAST**							
Cardioembolism, *n* (%)	54 (60.0)	34 (69.4)	20 (48.8)	0.08	25 (61.0)	29 (59.2)	1
**OCCLUSION SITE, *n* (%)**							
M1	49 (54.4)	24 (49.0)	25 (61.0)	0.06	18 (43.9)	31 (63.3)	<0.01
M2	15 (16.7)	6 (12.2)	9 (22.0)		4 (9.8)	11 (22.4)	
ICA	26 (28.9)	19 (38.8)	7 (17.1)		19 (46.3)	7 (14.3)	
**PROCEDURE RELATED FACTORS, MIN, MEDIAN (IQR)**							
LNT to recanalization	216.0 (142.0–317.0)	214.0 (154.0–316.0)	218.0 (134.0–317.0)	0.69	203.0 (152.0–315.0)	219.0 (134.0–336.0)	0.91
**MEAN BLOOD PRESSURE DURING 24 h AFTER ADMISSION, MEAN ± SD**							
SBP	128.8 ± 11.9	129.1 ± 10.3	128.5 ± 13.6	0.82	132.7 ± 10.1	125.5 ± 12.4	<0.01
DBP	73.3 ± 9.6	73.4 ± 10.6	73.2 ± 8.3	0.90	73.5 ± 9.0	73.1 ± 10.1	0.84
MBP	85.9 ± 8.9	86.0 ± 8.9	85.9 ± 9.0	0.97	87.1 ±7.4	84.9 ± 10.0	0.27
Parenchymal hemorrhage, n(%)	16 (17.8)	10 (20.4)	6 (14.6)	0.66	9 (22.0)	7 (14.3)	0.50
**EARLY NEUROLOGICAL IMPROVEMENT, *n* (%)**							
Day 1	40 (44.4)	16 (32.7)	24 (58.5)	0.03	7 (17.1)	33 (67.3)	<0.01
Day 7 or discharge	60 (66.7)	27 (55.1)	33 (80.5)	0.02	17 (41.5)	43 (87.8)	<0.01
**3 MONTH OUTCOME, *n* (%)**							
mRS ≤ 2	49 (54.4)	18 (36.7)	31 (75.6)	<0.01			
mRS ≤ 1	40 (44.4)	14 (28.6)	26 (63.4)	<0.01			
**COLLATERAL STATUS, *n* (%)**							
Poor collateral (0–3)					31 (75.6)	18 (36.7)	<0.01

*IQR, interquartile range; ER, emergency room; NIHSS, National Institutes of Health Stroke Scale; SD, standard deviation; DM, diabetes mellitus; HT, hypertension; Af, atrial fibrillation; CAD, coronary artery disease; TOAST, Trial of ORG 10172 in Acute Stroke Treatment; LNT, last –seen– normal time; SBP, systolic blood pressure; DBP, diatolic blood pressure; MBP, mean blood pressure; mRS, modified Rankin Scale; ICA, internal carotid artery*.

Antihypertensive medications during 24 h after admission were used in 39 patients (43.3%). Calcium channel blocker was the most frequently used medication (*n* = 35, 89.7%), followed by beta blocker (*n* = 4, 10.3%), and angiotensin II receptor blocker (*n* = 2, 5.1%). Four patients used 2 different kinds of antihypertensive medications. A delivery method of continuous intravenous infusion was used in 17 (43.6%) patients.

**Figure 1:**
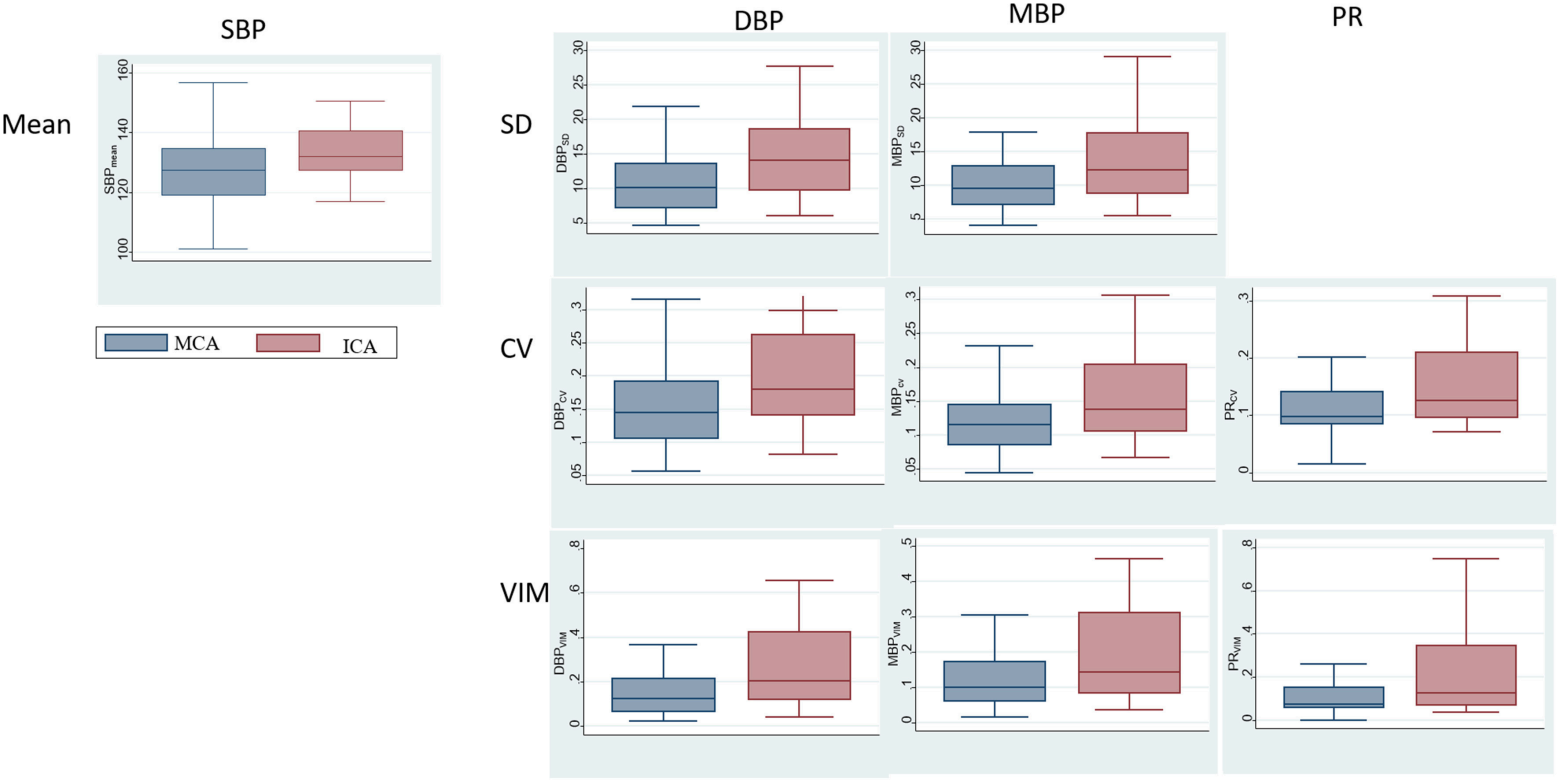
Comparison of blood pressure variability (BPV) parameters according to the occlusion sites.

**Figure 2:**
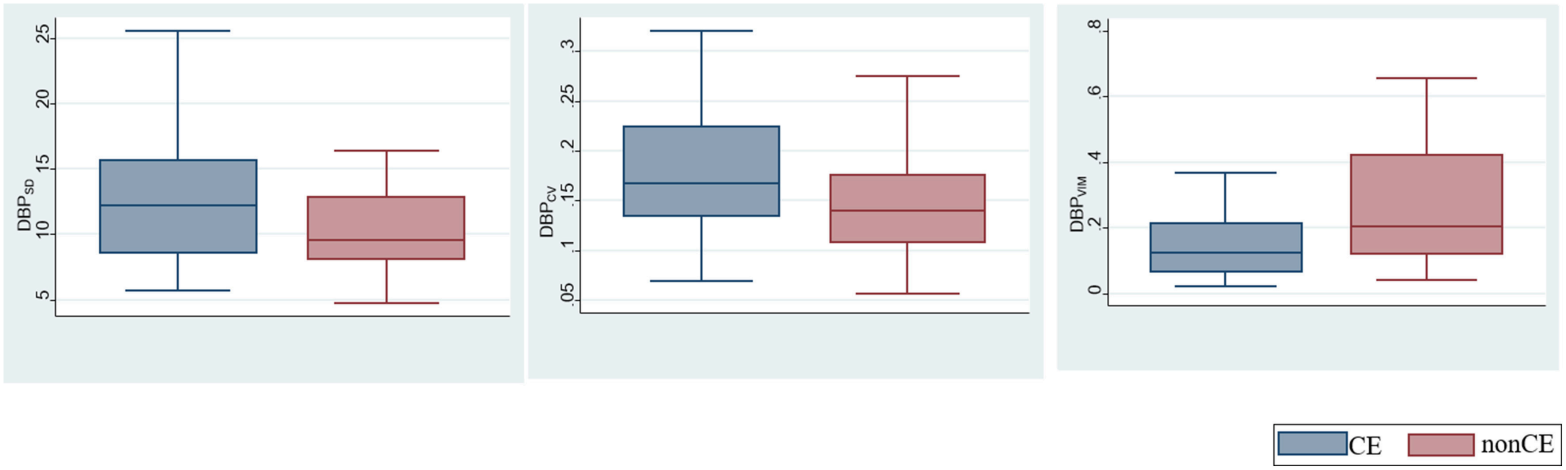
Comparison of blood pressure variability (BPV) parameters according to the mechanisms of stroke.

The comparisons of BPV parameters according to the occlusion sites (ICA vs. MCA) and mechanisms of stroke [cardioembolic (CE) vs. non-CE] are presented in Figure 1. Most BPV parameters [mean of SBP (SBP_mean_); SD, CV, and VIM of DBP (DBP_SD_, DBP_CV_, and DBP_VIM_) and MBP (MBP_SD_, MBP_CV_, and MBP_VIM_); and CV and VIM of PR (PR_CV_ and PR_VIM_)] were significantly higher in patients with ICA occlusion than in those with MCA occlusion (Figure 1, Table 2). DBP_SD_, DBP_CV_, and DBP_VIM_ were significantly higher in patients with CE stroke than in those with non-CE stroke (Figure 2, Table 2).

**Table 2:** Comparison of BPV parameters according to the occlusion site, mechanism of stroke, and recanalization time.

	**Occlusion site**			**Stroke mechanism**			
	**Total**	**MCA (*n* = 64)**	**ICA (*n* = 26)**	***P*-value**	**CE (*n* = 54)**	**Non-CE (*n* = 36)**	***P*-value**
SBP_mean_	128.80 ± 11.87	126.98± 12.29	133.27 ± 9.58	0.02	128.36 ± 11.36	129.45 ± 12.74	0.67
SBP_SD_	14.36 (11.79–20.14)	13.90 (11.36–18.44)	16.91 (13.44–25.34)	0.06	14.44 (10.80–20.14)	14.36 (11.99–19.91)	0.68
SBP_CV_	0.11 (0.09–0.14)	0.11 (0.09–0.13)	0.13 (0.09–0.19)	0.15	0.11 (0.09–0.14)	0.12 (0.09–0.15)	0.75
SBP_VIM_	0.11 (0.07–0.17)	0.10 (0.07–0.16)	0.13 (0.07–0.26)	0.21	0.10 (0.07–0.17)	0.11 (0.07–0.17)	0.78
DBP_mean_	73.29 ± 9.58	73.26 ± 10.22	73.39 ± 7.95	0.95	74.18 ± 9.40	71.97 ± 9.82	0.29
DBP_SD_	10.83 (8.33–14.75)	10.16 (7.23–13.59)	14.07 (9.82–18.67)	0.01	12.24 (8.57–15.66)	9.59 (8.09–12.83)	0.04
DBP_CV_	0.16 (0.11–0.20)	0.14 (0.11–0.19)	0.18 (0.14–0.26)	0.01	0.17 (0.13–0.22)	0.14 (0.11–0.18)	0.06
DBP_VIM_	0.14 (0.07–0.25)	0.12 (0.06–0.21)	0.20 (0.12–0.42)	0.01	0.16 (0.11–0.29)	0.12 (0.07–0.18)	0.05
MBP_mean_	85.92 ± 8.89	85.53 ± 9.68	86.87 ± 6.68	0.46	86.72 ± 8.36	84.69 ± 9.65	0.30
MBP_SD_	10.24 (7.89–13.93)	9.54 (7.28–12.80)	12.22 (8.85–17.78)	0.03	10.65 (7.56–15.28)	9.56 (8.08–11.62)	0.43
MBP_CV_	0.12 (0.10–0.16)	0.12 (0.09–0.14)	0.14 (0.11–0.20)	0.03	0.12 (0.09–0.18)	0.12 (0.10–0.13)	0.48
MBP_VIM_	0.10 (0.07–0.19)	0.10 (0.06–0.16)	0.14 (0.08–0.31)	0.03	0.11 (0.07–0.21)	0.10 (0.08–0.13)	0.52
PR_mean_	76.19 ± 14.89	77.00 ± 15.11	74.19 ± 14.43	0.42	76.54 ± 15.37	75.66 ± 14.34	0.79
PR_SD_	8.19 (6.08–11.11)	7.88 (5.98–10.61)	8.76 (6.17–14.57)	0.09	8.61 (6.07–12.08)	7.88 (6.19–10.68)	0.52
PR_CV_	0.11 (0.09–0.15)	0.10 (0.09–0.14)	0.13 (0.10–0.21)	0.03	0.12 (0.90–0.14)	0.10 (0.09–0.15)	0.42
PR_VIM_	0.09 (0.06–0.17)	0.08 (0.06–0.15)	0.12 (0.07–0.35)	0.09	0.11 (0.06–0.16)	0.08 (0.06–0.17)	0.42

*ICA, internal carotid artery; MCA, middle cerebral artery; CE, cardioembolism; non-CE, non-cardioembolism; SD, standard deviation; CV, coefficient of variation; VIM, variation independent of the mean; PR, pulse rate*.

Most of the BPV parameters (SBP_mean_, SBP_SD_, SBP_CV_, and SBP_VIM_; DBP_SD_, DBP_CV_, and DBP_VIM_; MBP_SD_, MBP_CV_, and MBP_VIM_; and PR_SD_, PR_CV_, and PR_VIM_) were significantly higher in patients with poor outcomes than in those with good outcomes among those with poor collateral circulation during the first 24 h after ERT. However, no significant differences were observed in these BPV parameters in patients with good collateral circulation based on functional outcomes (Table 3).

**Table 3:** Association of blood pressure variability parameters with clinical outcomes according to the collateral status.

	**Poor collateral**				**Good collateral**			
	**Total (*n* = 49)**	**Poor outcome (*n* = 31)**	**Good outcome (*n* = 18)**	***P*-value**	**Total (*n* = 41)**	**Poor outcome (*n* = 10)**	**Good outcome (*n* = 31)**	***P*-value**
SBP_mean_	129.06 ± 10.33	132.94 ± 9.29	122.38 ± 8.62	<0.01	128.48 ± 13.62	131.88 ± 12.86	127.38 ± 13.88	0.37
SBP_SD_	14.89 (11.84–20.95)	18.34 (14.44–23.72)	11.84 (10.72–12.95)	<0.01	14.02 (11.79–19.10)	13.94 (11.62–20.14)	14.02 (11.83–18.74)	0.94
SBP_CV_	0.12 (0.09–0.16)	0.14 (0.11–0.17)	0.09 (0.09–0.10)	<0.01	0.11 (0.09–0.14)	0.10 (0.09–0.15)	0.12 (0.09–0.14)	0.54
SBP_VIM_	0.11 (0.07–0.19)	0.15 (0.09–0.23)	0.07 (0.07–0.09)	<0.01	0.11 (0.06–0.16)	0.08 (0.06–0.17)	0.11 (0.07–0.15)	0.48
DBP_mean_	73.41 ± 10.60	72.50 ± 9.89	74.97 ± 11.85	0.44	73.16 ± 8.32	76.69 ± 4.56	72.02 ± 8.98	0.04
DBP_SD_	10.28 (8.33–15.09)	14.23 (9.74–17.17)	7.58 (6.52–10.28)	<0.01	11.14 (8.48–13.43)	9.12 (6.11–20.31)	11.14 (8.58–13.29)	0.57
DBP_CV_	0.16 (0.11–0.22)	0.20 (0.14–0.25)	0.11 (0.09–0.15)	<0.01	0.15 (0.11–0.19)	0.11 (0.08–0.26)	0.16 (0.12–0.18)	0.39
DBP_VIM_	0.14 (0.08–0.29)	0.24 (0.12–0.37)	0.07 (0.05–0.14)	<0.01	0.14 (0.07–0.19)	0.08 (0.04–0.42)	0.15 (0.09–0.19)	0.43
MBP_mean_	85.96 ± 8.91	86.14 ± 7.95	85.64 ± 10.60	0.85	85.87 ± 8.99	89.91 ± 4.60	84.53 ± 9.73	0.03
MBP_SD_	10.16 (7.94–13.93)	12.67 (9.46–16.96)	7.99 (5.73–9.54)	<0.01	10.37 (7.29–13.83)	10.06 (7.13–17.78)	10.37 (7.96–13.27)	0.74
MBP_CV_	0.12 (0.10–0.16)	0.14 (0.12–0.20)	0.10 (0.08–0.11)	<0.01	0.12 (0.09–0.15)	0.11 (0.08–0.20)	0.12 (0.10–0.14)	0.91
MBP_VIM_	0.12 (0.07–0.20)	0.17 (0.11–0.30)	0.07 (0.05–0.09)	<0.01	0.10 (0.07–0.17)	0.09 (0.04–0.28)	0.10 (0.07–0.15)	0.7
PR_mean_	77.79 ± 16.72	77.99 ± 15.57	77.43 ± 18.99	0.91	74.28 ± 12.30	79.68 ± 9.76	72.54 ± 12.66	0.11
PR_SD_	8.68 (6.09–12.08)	10.49 (7.02–14.57)	7.09 (5.20–8.24)	0.01	7.85 (6.08–9.35)	8.21 (7.72–8.82)	7.66 (5.77–10.11)	0.46
PR_CV_	0.12 (0.09–0.16)	0.13 (0.10–0.18)	0.09 (0.08–0.12)	<0.01	0.10 (0.09–0.14)	0.10 (0.09–0.13)	0.10 (0.08–0.14)	0.92
PR_VIM_	0.11 (0.06–0.20)	0.14 (0.08–0.327)	0.06 (0.04–0.11)	<0.01	0.08 (0.06–0.14)	0.08 (0.06–0.13)	0.08 (0.05–0.15)	0.92

*SD, standard deviation; CV, coefficient of variation; VIM, variation independent of the mean; PR, pulse rate*.

**Figure 3:**
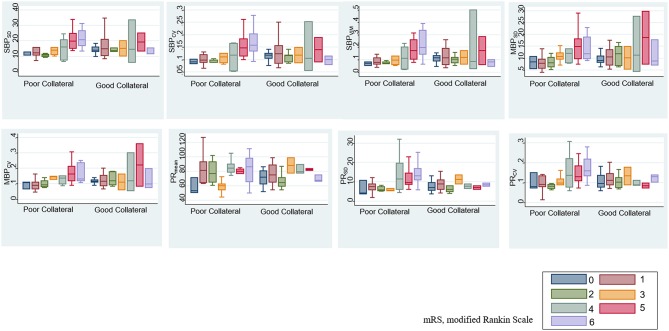
Association between BPV parameters and modified Rankin scale (mRS) based on baseline collateral circulation.

After adjustment for age, sex, initial severity of stroke (i.e., NIHSS), and occlusion site, the SBP_SD_, SBP_CV_, SBP_VIM_, and PR_mean_ remained significant predictors of ENI on day 1 and mRS at month 3 in patients with poor collateral circulation. MBP_SD_, MBP_CV_, and PR_SD_ were associated with mRS shift toward poor functional outcomes at month 3 (Table 4). Figure 3 indicates a positive association between the BPV parameters and mRS increment in the patients with poor collateral circulation.

**Table 4:** Association of blood pressure variability parameters with functional outcomes in poor collateral patients.

**BPV parameters**	**mRS at month 3 OR (95% CI)**	**ENI on day 1 OR (95% CI)**
SBP_SD_	2.20 (1.20–4.04)	0.22 (0.06–0.77)
SBP_CV_	2.18 (1.22–3.91)	0.22 (0.06–0.83)
SBP_VIM_	2.12 (1.19–3.75)	0.10 (0.01–0.88)
MBP_SD_	1.88 (1.01–3.52)	0.35 (0.11–1.15)
MBP_CV_	2.03 (1.06–3.87)	0.41 (0.13–1.24)
PR_mean_	1.50 (1.05–2.08)	0.48 (0.26–0.89)
PR_SD_	1.75 (1.01–3.05)	0.51 (0.22–1.19)

*Adjustment by age, sex, initial NIHSS, and occlusion site*.

*Odds ratio per 10 mmHg increment of mean and per 1 SD increment of SD, CV, and VIM*.

*Ordinal logistic regression for mRS at month 3; binary logistic regression for ENI on day 1*.

*BPV, blood pressure variability; SBP, systolic blood pressure; MBP, mean blood pressure; PR, pulse rate; SD, standard deviation; CV, coefficient of variation; VIM, variation independent of the mean; ENI, early neurological improvement; OR, Odds ratio; CI, confidence interval*.

No significant association between BPV and clinical outcomes were found after stratification based on the occlusion sites and stroke mechanisms.

## Discussion

As substantial reperfusion is achieved in 72–88% of cases currently (20), the importance of postreperfusion blood pressure management is increasing. Our study was intended to investigate the effect of postreperfusion BPV on clinical outcomes in ischemic stroke patients with successful recanalization. According to the result of recent study, increased BPV is associated with poor neurological outcome in patients with large ischemic core, large mismatch volume, proximal vessel occlusion, and good collaterals (11). Contrary to the prior study, the detrimental effect of increased BPV after recanalization was evident in patients with poor collateral circulation.

Collateral status is strongly correlated with initial stroke severity, baseline infarct volume, and the extent and speed of infarct growth (21). Patients with poor collateral flow present with more severe neurological status and experience the least benefit from the recanalization therapy because of the quick progression of the infarct volume (21). The ischemic core volume may be larger and peripheral vascular resistance may be greater in patients with poor collateral circulation than in those with good collateral circulation. Therefore, increased BPV may induce hemorrhagic transformation more frequently in patients with poor collateral circulation. Furthermore, BP fluctuation could induce a recurrent embolism from the proximal vasculature; the ability to wash out emboli may be reduced in patients with poor collateral circulation (22). Therefore, stroke progression or recurrence may occur more frequently in these patients.

The differences of BPV in patients with different occlusion sites and stroke subtypes are noteworthy. Although the absolute level of BP is not related to initial stroke severity and infarct volume (23, 24), higher BPV in patients with ICA occlusion suggests that these factors may have a correlation with BPV. More severe stroke is associated with the increased sympathetic activity, and sympathetic overactivity induces high BPV (16, 25, 26). Decreased function of arterial baroreceptor could also increase BPV (16, 26). Higher BPV in patients with ICA occlusion may be due to sympathetic overactivity and decreased baroreflex sensitivity. It is unclear why only DBP-related variables (DBP_SD_, DBP_CV_, and DBP_VIM_) are significantly higher in cardioembolic stroke than in other stroke types caused by different mechanisms including large artery atherosclerosis. Further studies are needed to assess whether the results are due to changes in coronary perfusion pressure affected by diastolic BPV or merely chance findings without clinical meaning.

There are several limitations to consider when interpreting our findings. First, the retrospective nature and small study population in this study warrant further investigation with a large, prospective, and multicenter cohort. Second, the association of BPV with clinical outcomes in patients with poor collateral circulation does not constitute a causal relationship. Because this study included a small number of hemorrhagic transformation or ischemic stroke recurrence, the mechanism of deleterious effects of BPV in patients with poor collateral status could not be determined. Lastly, pretreatment or intraprocedural blood pressure could also have influence on clinical outcome and obtaining these data retrospectively was not available and beyond the scope of the current analysis.

In conclusion, BPV during the first 24 h after recanalization had a greater impact on functional outcomes in patients with poor collateral circulation (i.e., pial arterial filling score <4). The results suggest that the close blood pressure management by decreasing BPV after recanalization might have effect on improving clinical outcomes in patients with acute large vessel occlusive stroke, particularly accompanied by poor collateral circulation. To increase clinical benefits of ERT, postreperfusion BP management, as well as expedited and complete recanalization, may be important in cases of poor collateral circulation among patients achieving successful recanalization after ERT.

## Ethics Statement

Seoul National University Bundang Hospital Institutional Review Board approved the retrospective study. Informed consent was waived for the retrospective chart review study.

## Author Contributions

JC and M-KH contributed to the study concept and design. JC, CJ, and DG contributed to the acquisition of data. JC and CJ contributed to the analysis and interpretation of data. JC and M-KH drafted the manuscript. S-BJ and M-KH performed critical revision of the manuscript for important intellectual content. M-KH provided the final approval of the final version of this manuscript. All authors agreed to be accountable for all aspects of the work in ensuring that questions related to the accuracy or integrity of any part of the work are appropriately investigated and resolved.

### Conflict of Interest Statement

The authors declare that the research was conducted in the absence of any commercial or financial relationships that could be construed as a potential conflict of interest.
